# Pregnenolone Inhibits Osteoclast Differentiation and Protects Against Lipopolysaccharide-Induced Inflammatory Bone Destruction and Ovariectomy-Induced Bone Loss

**DOI:** 10.3389/fphar.2020.00360

**Published:** 2020-03-27

**Authors:** Xiaochen Sun, Chenxi Zhang, Huan Guo, Jiao Chen, Yali Tao, Fuxiao Wang, Xixi Lin, Qian Liu, Li Su, An Qin

**Affiliations:** ^1^Guangxi Key Laboratory of Regenerative Medicine, Guangxi Medical University, Nanning, China; ^2^Institute of Translational Medicine, Shanghai University, Shanghai, China; ^3^Shanghai Key Laboratory of Orthopaedic Implants, Shanghai Ninth People’s Hospital, Shanghai Jiaotong University School of Medicine, Shanghai, China

**Keywords:** LPS (lipopolysaccharide), OVX model, osteoclast (OCs), ERK, RANKL (receptor activator of nuclear factor kappa-B ligand)

## Abstract

Osteolytic bone disease is characterized by excessive osteoclast bone resorption leading to increased skeletal fragility and fracture risk. Multinucleated osteoclasts formed through the fusion of mononuclear precursors are the principle cell capable of bone resorption. Pregnenolone (Preg) is the grand precursor of most if not all steroid hormones and have been suggested to be a novel anti-osteoporotic agent. However, the effects of Preg on osteoclast biology and function has yet to be shown. Here we examined the effect of Preg on receptor activator of nuclear factor kappa B ligand (RANKL)-induced osteoclast formation and bone resorption *in vitro*, and potential therapeutic application in inflammatory bone destruction and bone loss *in vivo*. Our *in vitro* cellular assays demonstrated that Preg can inhibit the formation of TRAP^+ve^ osteoclast formation as well as mature osteoclast bone resorption in a dose-dependent manner. The expression of osteoclast marker genes *CTSK*, *TRAP*, *DC-STAMP*, *ATP6V0d2*, and *NFATc1* were markedly attenuated. Biochemical analyses of RANKL-induced signaling pathways showed that Preg inhibited the early activation of extracellular regulated protein kinases (ERK) mitogen-activated protein kinase (MAPK) and nuclear factor-κB, which consequently impaired the downstream induction of c-Fos and NFATc1. Using reactive oxygen species (ROS) detection assays, we found that Preg exhibits anti-oxidant properties inhibiting the generation of intracellular ROS following RANKL stimulation. Consistent with these *in vitro* results, we confirmed that Preg protected mice against local Lipopolysaccharide (LPS)-induced inflammatory bone destruction *in vivo* by suppressing osteoclast formation. Furthermore, we did not find any observable effect of Preg on osteoblastogenesis and mineralization *in vitro*. Finally Preg was administered to ovariectomy (OVX)-induced bone loss and demonstrated that Preg prevented systemic OVX-induced osteoporosis. Collectively, our observations provide strong evidence for the use of Preg as anti-osteoclastogenic and anti-resorptive agent for the potential treatment of osteolytic bone conditions.

## Introduction

Osteolytic bone diseases such as inflammatory bone destruction and post-menopausal osteoporosis share a common theme of deterioration and destruction of the microstructure of bone tissue leading to marked decreases in bone mass, skeletal fragility, and consequently increased risk of bone fractures. The main cause of osteolytic diseases is the imbalance in the bone remodeling leading to excessive osteoclast formation and bone resorption. Osteoclasts are multinucleated giant cells formed through the fusion of mononuclear cells of the monocyte/macrophage cell lineage (Boyle et al., 2003). They are the principle and only cell in the body capable of resorbing and degrading the mineralized bone matrix (Lacey et al., 2012).

Two key cytokines are needed to stimulate the commitment of mononuclear precursor cells to the osteoclast lineage. One is the macrophage-colony stimulating factor (M-CSF) and the other one is the receptor activator of nuclear factor kappa B ligand (RANKL). M-CSF contributes to the proliferation and survival of early mononuclear precursors. RANKL on the other hand drives the differentiation and fusion of precursor cells toward the osteoclast lineage. Both M-CSF and RANKL is required for subsequent mature osteoclast activation toward bone resorption (Udagawa et al., 1990; Lacey et al., 1998; Yasuda et al., 1998).

Binding of RANKL to its receptor RANK initiates a cascade of early signaling events that culminates in the induction and sustained activation of the master osteoclast transcription factor NFATc1 that regulates a host of genes involved in osteoclast differentiation and bone resorption (Ishida et al., 2002; Takayanagi et al., 2002; Asagiri et al., 2005; Crotti et al., 2008; Aliprantis et al., 2008; Feng et al., 2009). Among the many RANKL-induced signaling cascades involved in the induction of NFATc1, the nuclear factor-κB (NF-κB) and mitogen-activated protein kinase (MAPK; ERK, p38, and JNK) signaling pathways, are considered the most important being activated early on in the differentiation process. Additionally, the level of reactive oxygen species (ROS) which are markedly elevated during osteoclast formation and bone resorption, also play important roles in the regulation of RANKL-induced signaling cascades (Garrett et al., 1990; Bhatt et al., 2002; Ha et al., 2004; Lee et al., 2005). The importance of these signaling molecules and transcription factors in osteoclast biology are exemplified by numerous genetic and pharmacological studies. Genetic deficiency of NF-kB, MAPK, c-Fos, and NFATc1 have led to osteopetrotic phenotypes in mice (Iotsova et al., 1997; Matsuo et al., 2000; Klein et al., 2006; Thouverey and Caverzasio, 2012), whereas pharmacological inhibition studies that impairs the activation of these signaling cascades show promise as therapeutic agents against osteolytic conditions (Zhang et al., 2018; Xiao et al., 2019; Wu et al., 2019).

Pregnenolone (Preg) is a natural endogenous steroid hormone and “grand” precursor of most if not all steroid hormones including estrogen, progesterone, testosterone, glucocorticoids, and mineralocorticoids (Henderson et al., 1950; Berg et al., 2002; Marx et al., 2011). Despite being the precursor steroid hormone, Preg and its metabolic derivatives have shown to exert anti-inflammatory, anti-cancer, and neuroprotective properties (Popovich et al., 2012; Elhinnawi et al., 2018; Cho et al., 2019). Furthermore, it has been suggested that Preg and heterocyclic analogues of Preg has potential as novel anti-osteoporotic agents by enhancing osteoblast differentiation and mineralization. However, the effects of Preg on osteoclast formation and bone resorption has yet to be investigated.

In this study, we showed that Preg can inhibit RANKL-induced osteoclastogenesis and bone resorption in a dose-dependent manner *in vitro* and protected mice against LPS-induced bone destruction and ovariectomy-induced bone loss *in vivo*. We found that Preg treatment was associated with the inhibition of early RANKL-induced activation of ERK MAPK and NF-κB signaling cascade which consequently attenuated the induction of c-Fos and NFATc1. Additionally, we also found Preg to exert anti-oxidant effects suppressing intracellular ROS production in response to RANKL. Collectively, our data suggests that Preg has potential as therapeutic agent against osteolytic conditions mediated by elevated osteoclast formation and bone resorption.

## Materials and Methods

### Media and Reagents

Preg was purchased from Tokyo Chemical Industry (TCI Development, Shanghai, China, purity>98%), dissolved in dimethyl sulfoxide (DMSO; Beyotime Institute of Biotechnology, Jiangsu, China) at stock concentration of 20 mM, and further diluted in culture media to working concentrations prior to use. Alpha modification of minimal essential medium (α-MEM) was from Hyclone (GE Healthcare, Chicago, IL, USA). Fetal bovine serum (FBS), L-glutamine, and penicillin and streptomycin (P/S) were obtained from Gibco (Thermo Fisher Scientific, Waltham, MA, USA). Recombinant mouse macrophage-colony stimulating factor (M-CSF) and receptor activator of nuclear factor-κB ligand (RANKL) were procured from R&D Systems (Minneapolis, MN, USA). Tartrate-resistant acid phosphatase (TRAP) staining kit acquired from Joytech Bio Inc (Zhejiang, China). Specific primary antibodies against NF-κB p65 (#4764), p-p65 (Ser536; #3033), IκBα (#4812), ERK1/2 (#4695), p-ERK1/2 (Thr202/Tyr204; #4370), p38 (#8690), p-p38 (Thr180/Tyr182; #4511), SAPK/JNK (#9252), p-SAPK/JNK (Thr183/Tyr185; #4668), β-actin (#4970), and GAPDH (#5174) were purchased from Cell Signaling Technology (Danvers, MA, USA). Fluorescently-labeled secondary goat anti-rabbit IgG antibody (IRDye 800CW; ab216773), Phalloidin-iFluor 488 Reagent (ab176753), and specific primary antibody against c-Fos (ab190289) were obtained from Abcam (Cambridge, UK). Specific primary antibody against NFATc1 (7A6; sc-7294) was from Santa Cruz Biotechnology (Dallas, TX, USA). 2-(4-Amidinophenyl)-6-indolecarbamidine (DAPI; C1002), ROS Assay Kit (S0033), and 5-bromo-4-chloro-3-indolyl phosphate (BCIP)/nitro blue tetrazolium (NBT) Alkaline Phosphatase Color Development Kit (C3206) were purchased from Beyotime Institute of Biotechnology. Alizarin Red S Solution (1%, pH4.2; G1452) was procure from Solarbio Life Sciences (Beijing, China). Prime Script RT Master Mix (#RR036A) and TB Green Premix Ex Taq (RR420A) were from Takara Bio Inc. (Shiga Prefecture, Japan).

### Bone Marrow-Derived Macrophages (BMMs) and Bone Marrow-Derived Stroma Cells (BMSCs) Isolation

Mouse BMMs and BMSCs were isolated by flushing the tibias and femurs of 6-week old mice with α‐MEM supplemented with 10% FBS, 2 mM L‐glutamine and 1% P/S (complete α-MEM). BMMs were maintained in complete α-MEM containing 30 ng/ml M-CSF. BMSCs were maintained in α-MEM supplemented with 15% FBS, 2 mM L-glutamine, and 1% P/S. All cells were grown in a 37°C incubator with humidified atmosphere of 95% air and 5% CO_2_, and the medium was replaced every 2–3 d. Adherent cells were grown to 95% confluence and were either passaged or used for downstream applications.

### Cell Viability Assay

Cell viability of BMMs and BMSCs were examined using the CCK-8 Cell Proliferation Assay Kit (Dojindo Molecular Technology, Kumamoto, Japan) in accordance with manufacturer’s protocol. The assay is based on the principle that the highly water-soluble tetrazolium salt, WST-8 [2-(2-methoxy-4-nitrophenyl)-3-(4-nitrophenyl)-5-(2,4-disulfophenyl)-*2H*-tetrazolium, monosodium salt] can be reduced to a water-soluble formazan dye by dehydrogenases in cells. The amount of formazan dye generated is directly proportional to the number of live cells. M-CSF-dependent BMMs or BMSCs were seeded in 96-well culture plates at a density of 6×10^3^ cells/well and allowed to adhere overnight. Next day, cells were treated without or with various concentrations (2.5, 5, 10, and 20 μM) of Preg for 48 and 96 h after which 10 μl of CCK-8 reagent was added to each well and incubated for further 4 h. After incubation, the absorbance was measured at wavelength of 450 nm on a spectrophotometer microplate reader (SpectraMax M2, Molecular Devices, SV, USA) and the average OD values for each sample were analyzed using ImageJ software (NIH, Bethesda, MD, USA).

### *In Vitro* Osteoclast Formation and Bone Resorption Assay

For osteoclast formation assays, BMMs seeded in 96-well plates at a density of 8×10^3^ cells/well in complete α-MEM containing 30 ng/ml M-CSF and 50 ng/ml RANKL were treated without or with various concentrations (5–20 μM) of Preg for 7 d. Medium containing M-CSF, RANKL, and Preg were changed every 2 d. At the end of 7 d, cells were fixed with 4% paraformaldehyde (PFA) for 20 min and stained for TRAP activity. TRAP stained cells were imaged under light microscopy and the number and size (in terms of area) of TRAP^+ve^ multinucleated osteoclasts with three or more nuclei were quantified using ImageJ software. For bone resorption assays, BMMs were cultured in six-well plates with 30 ng/ml M-CSF and 50 ng/ml RANKL for 3–4 d until small pre-osteoclast-like cells formed. Removed the cells with trypsin for 3 min and gently scraped them off and then centrifuge at 1,000 rpm for 5 min. Then equally reseed the cells onto hydroxyapatite-coated plates. Let the cells settle for 8 h, then treated with various concentrations (5, 10, and 20 μM) of Preg. After 4 d, adherent cells were incubated with 5% sodium hypochlorite solution for 15 min and then washed three times with PBS. Plates were air-dried and resorption pits visualized under light microscopy. The percentage of resorbed area relative to total well area for each experimental condition were measured using ImageJ software.

### *In Vitro* Osteogenic Differentiation, and Alkaline Phosphatase (ALP) and Mineralization Activity

BMSCs seeded in 12-well plates at a density of 3×10^5^ cells/well and grown to 90% confluence were then cultured in osteogenic medium containing 100 μM L-ascorbic acid, 10 mM β-glycerophosphate, and 100 nM dexamethasone without or with 10 or 20 μM for 7 d to induce osteogenic differentiation. Half osteogenic medium change was carried out every 2 d. At the end of the 7^th^ day, cells were fixed in 4% PFA for 20 min and then stained for ALP activity using the BCIP/NBT ALP Color Development Kit as per manufacturer’s protocol. For the examination of bone mineralization activity, BMSCs were cultured in osteogenic medium and treated as described above for 21 d with half medium change every 2 d. At the end of the experimental period, cells were fixed in 4% PFA for 20 min at room temperature, washed three times with 70% alcohol, and then stained with 1% Alizarin Red S solution at room temperature for 30 min. Plates were air-dried before imaging under light microscopy. ALP and mineralization activity (expressed as percentage of control) were measured using ImageJ software.

### RNA Extraction and Real-Time qPCR

Osteoclasts were generated from M-CSF-dependent BMMs after 7 d of culture with 50 ng/ml RANKL in the absence or presence of various concentration (5–20 μM). At the end of the culture period, total RNAs were extracted using TRIzol reagent (Thermo Fisher Scientific) as per manufacturer’s instructions. Complementary DNAs (cDNAs) were reversed transcribed using 1 μg of extracted total RNAs using Prime Script RT Master Mix and used as template for subsequent real time qPCR reactions. Real time qPCR was carried out on a qTOWER Real-time PCR Thermal Cycler (Analytik Jena, Jena, Germany) in reaction mixtures containing TB Green Premix Ex Taq, cDNA, and forward and reverse primers. The reaction conditions were 95°C for 3 min; followed by 40 cycles at 95°C for 10 s, 60°C for 20 s, and 72°C for 20 s; with a final extension step at 72°C for 20 s. The following primer sets based on mouse gene sequences were used: GAPDH (Forward:5'-ACCCAGAAGACTGTGGATGG-3', Reverse: 5'CACATTGGGGGTAGGAACAC-3'); *CTSK* (Forward: 5'-CTTCCAATACGTGCAGCAGA-3', Reverse: 5'-TCTTCAGGGCTTTCTCGTTC-3'); *TRAP* (Forward: 5'-CTGGAGTGCACGATGCCAGCGACA-3', Reverse: 5'-TCCGTGCTCGGCGATGGACCAGA-3'); *NFATc1* (Forward: 5'-CCGTTGCTTCCAGAAAATAACA-3', Reverse: 5'-TGTGGGATGTGAACTCGGAA-3'); *ATP6V0d2* (Forward: 5'-AAGCCTTTGTTTGACGCTGT-3', Reverse: 5'-TTCGATGCCTCTGTGAGATG-3'); and *DC-STAMP* (Forward: 5'-AAAACCCTTGGGCTGTTCTT-3', Reverse: 5'-AATCATGGACGACTCCTTGG-3'). Data were normalized to GAPDH using 2^−ΔΔCT^ method.

### Protein Extraction and Western Blot Analyses

To investigate early RANKL-induced signaling events, seeded BMMs were serum starved for 1 h, pre-treated without or with 20 μM Preg for 3 h, and then stimulated with 50 ng/ml RANKL for 5, 10, 20, 30, or 60 min. Unstimulated cells were used as time 0 mock controls. At the end of the experimental period, cells were lysed in cell lysis buffer containing radioimmunoprecipitation, protease and phosphatase inhibitor cocktail (Thermo Fisher Scientific) and cleared by centrifugation. For western blot, 30 μg of cleared protein lysates were resolved by sodium dodecyl sulfate‐polyacrylamide gel electrophoresis (SDS-PAGE) and then transferred to nitrocellulose membranes using Trans-Blot Turbo Transfer System (Bio-Rad Laboratories, Hercules, CA, USA). Membranes were blocked with 5% skim milk for 1 h and then washed three times with TBST (Tris-buffered saline with 0.1% Tween 20). Membranes were incubated with specific primary antibody of interest (diluted 1:1,000) overnight at 4°C followed by incubation with appropriate fluorescently-labeled secondary antibody (IRDye; diluted 1:1,000) for 1 h at room temperature. Protein bands were visualized using the Odyssey Imaging System (LI-COR Biosciences, Lincoln, NE, USA) and relative protein expression calculated from gray-scale blots using ImageJ software.

### Intracellular ROS Assay

Intracellular ROS levels were detected using the ROS assay kit containing the cell-permeant fluorogenic dye, 2’,7’‐dichlorodihydrofluorescein diacetate (DCFH-DA). Upon entering cells, DCFH-DA is deacetylated to a non-fluorescent compound and then under elevated ROS conditions, is oxidized to highly fluorescent 2’,7’‐dichlorofluorescein (DCF). M-CSF-dependent BMMs seeded in 96-well plates at a density of 8×10^3^ cells/well were stimulated with 50 ng/ml RANKL without or with 10 or 20 μM Preg for 3 d. At the end of 3 d, cells were incubated with 10 μM DCFH-DA for 40 min in the dark and DCF fluorescence visualized under a fluorescence microscope (Nikon Corporation, Tokyo, Japan). ROS levels were quantified by ImageJ software.

### Flow Cytometric Analysis

BMMs were seeded in six-well plate at a density of 3×10^5^ cells/well were stimulated with 50 ng/ml RANKL without or with 10 or 20 μM Preg for 3 d. At the end of 3 d, cells were incubated with 10 μM DCFH-DA for 40 min in the dark and detected with flow cytometry.

### Cytoskeletal F-Actin Podosomal Belt Staining

M-CSF-dependent BMMs seeded in 96-well plates at a density of 8×10^3^ cells/well and were stimulated with 50 ng/ml RANKL without or with 10 or 20 μM Preg for 7 d or until matured multinucleated osteoclasts formed. Cells were then fixed in 4% PFA for 20 min, permeablized with 0.5% Triton X-100 in PBS for 5 min, and then incubated with Phalloidin-iFluor 488 reagent in 1% BSA-PBS for 90 min at room temperature. Nuclei were counterstained with DAPI (5 μg/ml) for 5 min. Fluorescence images were captured under fluorescence microscopy.

### Animal Ethics Statement

All animal experiments and models were approved and performed in accordance with the guidelines of the Animal Care Committee of Guangxi Medical University and the Guide for the Care and Use of Laboratory Animals of the National Institute Health (USA). All animals were housed in temperature controlled environment of 22–25°C with 12 h light/dark cycle and fed standard rodent chow and water *ad libitum*.

### *In Vivo* LPS-Induced Calvarial Osteolysis Model

Twenty-four 8-week-old male C57BL/6 mice were randomly assigned into four groups (n = 6 mice per group): sham control (mock operation with PBS injection), LPS only (5 mg/kg bodyweight), LPS with low dose Preg (1 mg/kg bodyweight), and LPS with high dose Preg (10 mg/kg bodyweight). Mice were anesthetized *via* the use of isoflurane gas, and then all mice received subcutaneous injection over the sagittal midline suture of the calvarium. On day 0, sham control and LPS group received PBS or LPS and PBS injection respectively, whereas Preg treatment groups received LPS and Preg injections together. PBS and Preg was injected every other day over a 7 d period after which all mice were sacrificed. Calvarial bones were harvested, fixed in 4% PFA and processed for micro-CT and histological assessment. All mice behaved normally with no adverse effects or unexpected fatalities were observed throughout the experimental period.

### *In Vivo* Ovariectomy (OVX)-Induced Bone Loss Model

Twenty-four 12-week-old female C57BL/6 mice were randomly allocated into four groups (n = 6 per group): sham control (mock operation with PBS injection), OVX group (with PBS injection), OVX with low-dose Preg (1 mg/kg bodyweight), and OVX with high-dose Preg (10 mg/kg bodyweight). Under isoflurane gas anesthesia, all mice except those in the sham control groups underwent bilateral ovariectomy to remove ovaries and fallopian tubes were ligated in order to induce bone loss and bone architectural deterioration. Mice in sham control group underwent surgery but ovaries were not removed. All mice were allowed one week of post-operative recovery prior to the commencement of treatment. All mice mice were intraperitoneally injected with PBS (sham and OVX groups) or with Preg (low- or high-dose groups) every 2 d over 4 weeks. After 4 weeks of treatment, all mice were sacrificed by cervical dislocation and tibias were removed, fixed in 4% PFA for 2 d and then processed for micro-CT scan and histological assessments.

### Micro-Computed Tomography (μCT) and Histological Assessment

Three-dimensional reconstructions of the harvested bone samples (calvarias and tibias) were created from images acquired using the Skyscan 1176 high-resolution micro-CT scanner (Bruker; Billerica, MA, USA). For calvarial bone samples, image acquisitions were carried out at a voltage of 45 kV, a current of 550 μA, and isotropic resolution of 18 μm. For tibial bone samples image acquisitions parameters were a voltage of 50 kV, current of 450 μA, and isotropic resolution of 9 μm. A 0.5–0.75 mm thick aluminum filter was used for beam-hardening reduction. Quantitative morphometric analyses were carried out with a square region of interest (ROI) around the midline suture of calvarial bones or 0.5 mm below the tibial growth plate. Bone parameters analyzed for calvarial bone samples include bone volume/total volume (BV/TV) and percentage of porosity. For tibial bone samples BV/TV, bone surface to total volume ratio (BS/TV, mm^−1^), trabecular spacing (Tb.Sp, mm), and trabecular number (Tb.N, mm^−1^) were analyzed. Following μCT scanning, the fixed bone samples were decalcified in 10% EDTA for 2 weeks, and then embedded in paraffin blocks for sectioning into 5 μm thick sections. Sections were stained with hematoxylin-eosin or for TRAP activity, and imaged at 40X and 100X magnification under a light microscope (Nikon, Tokyo, Japan).

### Statistical Analyses

All data in this study are presented as mean ± standard deviation or representative images of at least three independent experiments performed in triplicates unless otherwise specified. Statistical analyses were performed using SPSS 19.0 software (IBM Corporation, New York, NY, USA) adopting Student’s *t*-test or one-way analysis of variance (ANOVA). P values less than 0.05 or otherwise indicated were considered statistically significant.

## Results

### Preg Inhibited RANKL-Induced Osteoclastogenesis

**Figure 1:**
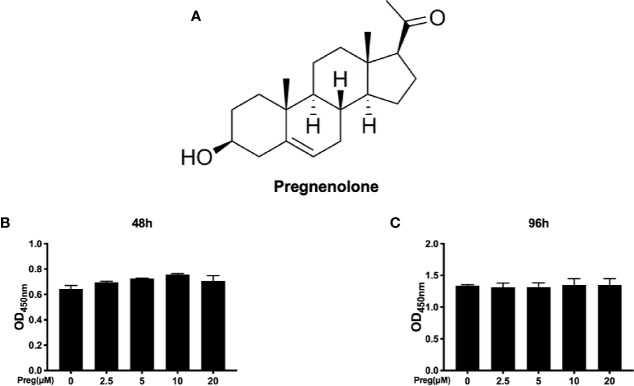
Cytotoxicity of pregnenolone (Preg) on osteoclast precursor cells **(A)** Chemical structure of Preg. **(B**, **C)** BMM cell viability as assessed by CCK-8 assay following treatment without or with indicated concentrations of Preg for **(B)** 48 and **(C)** 96 h. Data presented as mean ± standard deviation (n = 3).

Before we assess the effects of Preg (**Figure 1A**) on RANKL-induced osteoclast formation, we first established potential cytotoxic effects on BMM precursor cells using the CCK-8 viability assay. BMMs were treated with different concentrations of Preg for 48 and 96 h prior to incubation with CCK-8 reagent. As shown in **Figures 1B, C**, the viability of BMMs did not change after treatment with Preg for 48 and 96 h, indicating that Preg is not toxic to osteoclast precursor cells at concentrations up to 20 μM.

**Figure 2:**
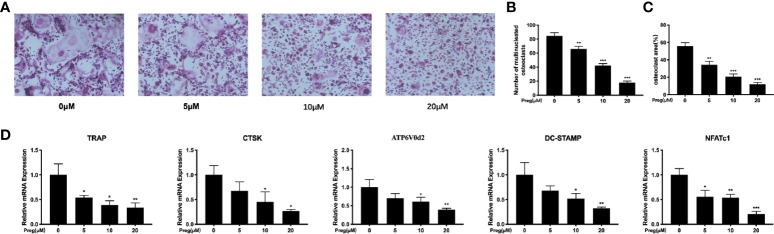
Pregnenolone (Preg) dose-dependently inhibits RANKL-induced osteoclast differentiation and gene expression *in vitro*. **(A)** Representative TRAP stained images of BMM-derived osteoclasts stimulated with RANKL for without or with indicated concentrations of Preg. The **(B)** number and **(C)** size (cell spread area) of TRAP^+ve^ multinucleated osteoclasts with three or more nuclei were quantified. **(D)** The relative expression of osteoclast marker genes (*TRAP, CTSK, DC-STAMP, NFATc1, ATP6V0d2*, and *c-Fos*) following Preg treatment were quantified by real time PCR. Values presented as the mean ± standard deviation (n = 3); *p < 0.05, **p < 0.01, ***p < 0.001.

Having now established that Preg does not affect BMM cell viability, we next examined the effect of Preg on RANKL-induced osteoclast formation *in vitro*. M-CSF-dependent BMMs were stimulated RANKL in the absence or presence of different concentrations of Preg for 7 d. As shown in **Figures 2A, B**, a dose-dependent decrease in the total number of TRAP^+ve^ multinucleated osteoclasts was observed following treatment with Preg. The number of TRAP^+ve^ osteoclasts decreased from 84 ± 3 in untreated controls to around 17 ± 2 in group treated with 20 μM of Preg (p < 0.0001). The lowest concentration of Preg that significantly inhibited osteoclast formation was found to be 5 μM. Furthermore, the size or cell spread area of osteoclasts that managed to form under the inhibitory effects of Preg were similarly dose-dependently reduced (**Figure 2C**), indicating potential adverse effect on precursor cell fusion and/or cytoskeletal alterations. Consistent with these cellular effects, analysis of the expression of osteoclast marker genes showed a similar trend (**Figure 2D**). The expression of genes involved in osteoclast differentiation and precursor cell fusion such as *NFATc1*, and *DC-STAMP* were significantly suppressed in the presence of Preg treatment. Similarly, the expression of genes involved in the bone resorptive process such as *TRAP* and *CTSK* also dose-dependently reduced. The gene *ATP6V0d2* which encodes the d2 subunit of the V-ATPase proton pump which has been implicated to be important for both precursor cell fusion and mature osteoclast bone resorption (Lee et al., 2006; Kim et al., 2008; Wu et al., 2009; Crasto et al., 2013; Takagi et al., 2017) showed a trend of decrease following Preg treatment. Collectively these results indicates that at the concentrations tested, Preg possess dose-dependent anti-osteoclastogenic effects with little adverse effects on cell viability.

### Preg Impaired Osteoclast Fusion and Bone Resorption *In Vitro*

**Figure 3:**
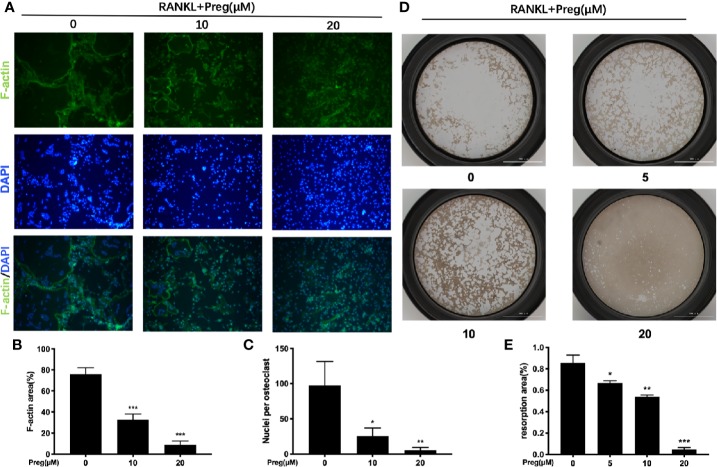
Pregnenolone (Preg) attenuated RANKL-induced osteoclast fusion and bone resorption. **(A)** Representative fluorescence images of actin stained BMM-derived osteoclasts stimulated with RANKL for 7 d with RANKL without or with 10 and 20 μM Preg. Actin cytoskeleton were stained Phalloidin-iFluor 488 (green) and nuclei with DAPI (blue). The **(B)** average cell size (cell spread area based on actin podosomal belt) and **(C)** average number of nuclei per osteoclasts were quantified. **(D)** Representative images of bone resorption by mature osteoclasts in the absence or presence of Preg. Pre-osteoclasts stimulated with RANKL for 3 d were reseeded onto hydroxyapatite-coated OsteoAssay plates and then treated without or with indicated concentrations of Preg for further 3 d. Cells were removed with sodium hypochlorite, and resorption pits were imaged under light microscopy. **(E)** The resorption area expressed as a percentage of total well area for each condition were quantified. Values presented as the mean ± standard deviation (n = 3); *p < 0.05, **p < 0.01, ***p < 0.001.

As Preg treatment affects the spreading of the osteoclasts we thus examined the integrity of the actin cyskeleton. The formation of a podosomal actin “belt” around the individual osteoclasts is a characteristic feature of well-spread multinucleated osteoclast, as seen in the RANKL-only treated cells (**Figure 3A**). Consistent with previous TRAP stained results, treatment with Preg significant reduced osteoclast cell spread area, with Preg-treated osteoclasts markedly smaller than untreated controls (**Figure 3B**). Additionally, examination of the DAPI-stained nuclei in Preg treated and untreated groups, we observed a striking reduction in the average number of nuclei per osteoclasts (**Figure 3C**). In fact, cells treated with 10 and more so with 20 μM of Preg were of mononuclear in nature as compared to the multinucleated giant cells in untreated controls. These data combined with previous gene expression results strongly indicates that monocytic precursor cell fusion in response to RANKL is substantially arrested following Preg treatment.

The ability to condense and form an intact cytoskeletal podosomal actin belt is a necessary perquisite for subsequent polarization of osteoclast toward bone resorption. Hence we next examined whether the bone resorptive function of mature osteoclast were impaired following Preg treatment. Osteoclasts were cultured on bone-mimicking hydroxyapatite coated substrate plates in the absence or presence of indicated concentrations of Preg. As shown in **Figure 3D**, a dose-dependent reduction in bone resorption was seen. Compared with untreated controls which shows extensive clearing of the hydroxyapatite substrate, osteoclasts treated with 20 μM of Preg exhibited almost no bone resorption (reduction of about 95%) with small pits scattered around the well (**Figures 3D, E**). No significant difference was observed between osteoclasts treated with the lowest concentration of Preg (5 μM) and with untreated controls. Approximately 25% and 35% reduction was seen in osteoclasts treated with 5 and 10 μM Preg respectively (**Figure 3E**). Thus at high doses, Preg not only inhibits osteoclast formation, but also bone resorption function mediated by mature osteoclasts.

### Preg Suppressed RANKL‐Induced ROS Level *In Vitro*

**Figure 4:**
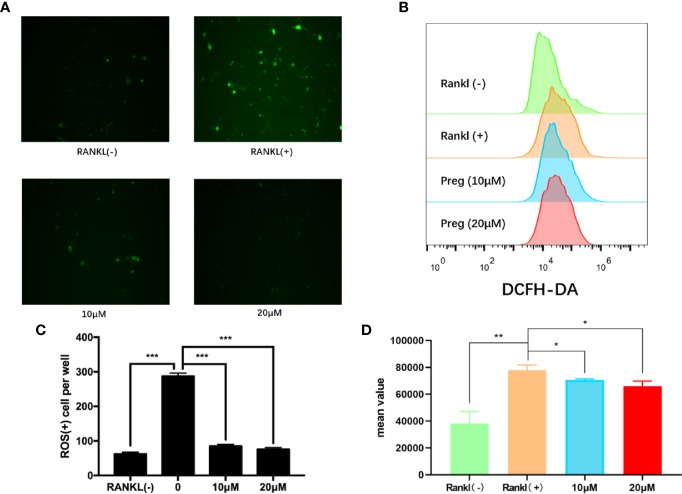
Pregnenolone (Preg) suppressed RANKL-induced intracellular ROS production. **(A, B)** BMMs were stimulated with RANKL in the absence or presence of indicated concentrations of Preg for 3 d and intracellular ROS was detected by the intracellular conversion of non-fluorescent DCFH-DA to highly fluorescent DCF. BMMs stimulated with M-CSF only was used as negative control. **(C)** The number of ROS positive cells were quantified. **(D)** The mean value of DCFH-DA were calculated. Values presented as the mean ± standard deviation (n = 3); *p < 0.05, **p < 0.01, ***p < 0.001.

Preg is the precursor or metabolic intermediate in the biosynthesis of most if not all steroid hormones including estrogens (Hu et al., 2010; Miller and Auchus, 2011). Estrogens are important steroid hormones in the regulation of both osteoblast and osteoclast formation and activity ((Novack, 2007; Imai et al., 2010). Furthermore, estrogens have been documented to possess potent anti-oxidant properties (Sack et al., 1994; Arnal et al., 1996; Sudoh et al., 2001; Lean et al., 2003; Reyes et al., 2006) and elevated ROS have been shown to induce osteoclast formation and activity(Garrett et al., 1990; Bax et al., 1992; Callaway and Jiang, 2015). Hence as a precursor to estrogen and its derivatives we investigated whether Preg has potential anti-oxidant effects against elevated ROS during the process of osteoclastogenesis. As shown in **Figure 4A**, the marked elevation in intracellular ROS levels following stimulation with RANKL was drastically inhibited following treatment with 10 and 20 μM Preg. The level of intracellular ROS in Preg treated cells were comparable to basal levels in untreated BMMs (**Figure 4C**). In order to confirm the inhibitory effect of Preg on intracellular ROS after RANKL stimulation, we also verified this by flow cytometry. The results showed that RANKL stimulation significantly increased the intracellular ROS level (**Figure 4B**). After treatment with 10 and 20 μM, There is a downward trend, but it is still higher than control cells (**Figure 4D**). Thus the data suggests that Preg may possess anti-oxidant properties.

### Preg Attenuated the RANKL Downstream Activation of ERK/c-fos/NFATc1 Signaling Pathway

**Figure 5:**
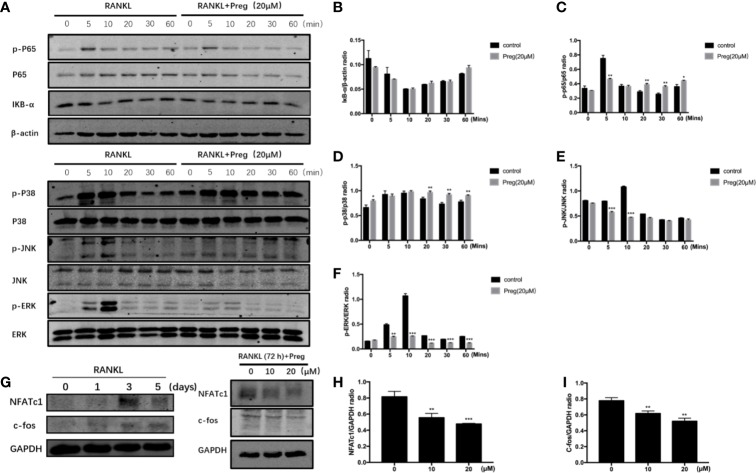
Pregnenolone (Preg) inhibited the RANKL-induced activation of ERK and NF-κB signaling cascades, and the downstream induction of c-Fos and NFATc1. **(A)** To examine early RANKL signaling events, BMMs were serum-starved for 1 h, pre-treated with 20 μM Preg for 3 h, and then stimulated with RANKL for the indicated time. Total cellular protein were extracted and subjected to western blot analyses using specific antibodies against ERK and p-ERK, p38 and p-p38, JNK and p-JNK, IκBα, NF-κB p65 and p-p65, and β-actin. **(B–F)** Quantitative densitometric analysis of phosphorylated protein to total protein counterpart, or normalized to β-actin were conducted. **(G)** To examine downstream (late stage) RANLK signaling events, BMMs were treated with RANKL for 0, 1, 3, and 5 d. M-CSF-dependent BMMs were stimulated with RANKL without or with 10 and 20 μM Preg for 72 h. Total cellular proteins were extracted and subjected to western blot analyses using specific antibodies against NFATc1, c-Fos, and GAPDH. **(H, I)** Quantitative densitometric analysis of (h) NFATc1 and (i) c-Fos normalized to GAPDH were conducted. Values presented as the mean ± standard deviation (n = 3); *p < 0.05, **p < 0.01, ***p < 0.001.

Osteoclast differentiation requires the timely and coordinated activation of various signaling pathways in response to RANKL stimulation. To determine the potential signaling pathways affected by Preg treatment, we examined early activation of two key RANKL-responsive signaling pathways, the NF-kB, and MAPK signaling cascades. As shown in the **Figures 5A, B**, the early activation of ERK MAPK as determined by the phosphorylation of ERK in response to RANLK was significantly attenuated following treatment with Preg. The activation phosphorylation of the other MAPK members, p38, and JNK was not affected by Preg treatment (**Figures 5A, C, D**). In terms of the early activation of the NF-κB signaling cascade, we only observed inhibition of IκBα degradation at the 5 and 60 min time points, whereas p65 phosphorylation was only inhibited at the 5 min time point (**Figures 5A, E, F**).

The timely and coordinated activation of NF-κB and MAPK is necessary for the subsequent induction of c-Fos and NFATc1. NFATc1 is a crucial transcription factor required for precursor cell fusion and terminal osteoclast differentiation by transcriptionally regulating the expression of numerous osteoclast genes. We first examined the expression of NFATc1 during osteoclast formation. We found the expression of NFATc1 peaks at day 3. So we investigated the effect of Preg on NFATc1 and c-Fos on day 3. The expression of c-Fos and NFATc1 was induced 72 h after RANKL stimulation, but was drastically reduced when cells were cultured in the presence of Preg in a dose-dependent manner (**Figures 5G–I**). Together our biochemical analyses suggest that Preg inhibits osteoclast formation in part by suppressing intracellular ROS production and attenuating RANKL-induced activation of ERK MAPK and NF-κB signaling cascades which subsequent reduced the effective downstream induction of Fos and NFATc1.

### Preg Protects Against LPS-Induced Osteoclast-Mediated Bone Loss *In Vivo*

**Figure 6:**
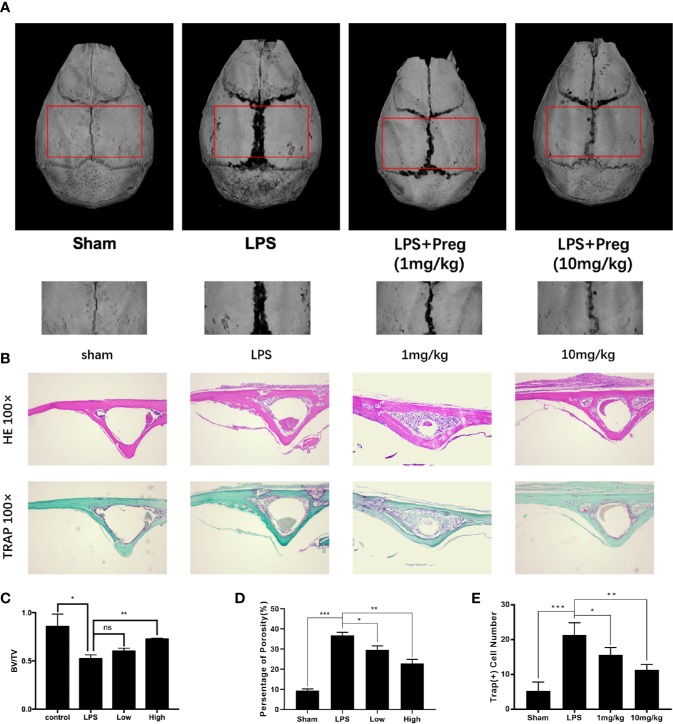
Pregnenolone (Preg) protects against LPS-induced inflammatory osteolysis of mouse calvarium *in vivo*. **(A)** Representative 3D μCT reconstructions of mouse calvarium from each treatment group. All subcutaneous injections were conducted over the sagittal midline suture of the calvarium. sham and LPS (5 mg/kg body weight) received injections of PBS and LPS respectively. Preg treatment groups received injections of LPS and Preg (low—1 mg/kg or high—10 mg/kg) together. Injection were carried out every day over a 7 d period after which all mice were sacrificed. **(B)** Representative hematoxylin-eosin staining (H&E) (100X magnification) and TRAP (100X magnification) stained sections from each treatment group. Calvarial bones were decalcified in 10% EDTA for 2 weeks, embedded in paraffin for histological sectioning, and then stained with H&E and TRAP. **(C–E)** Quantitative analysis of **(C)** bone volume to total tissue volume (BV/TV), **(D)** percentage of porosity, and **(E)** the number of TRAP^+ve^ cells were quantified. Values presented as the mean ± standard deviation (n = 3); *p < 0.05, **p < 0.01, ***p < 0.001.

Next, we investigated whether the promising *in vitro* cellular effect of Preg on osteoclast formation and bone resorption can be translated to protective outcomes against osteoclast-mediated bone destruction *in vivo*. LPS-induced calvarial bone destruction model to mimic inflammatory bone destruction. 3D reconstructions of the calvarial bone showed that when compared to sham controls, extensive bone destruction, loss of bone volume, and increased bone porosity around the midline suture of the calvarium was observed 7 d after the administration of LPS (**Figures 6A, C, D**). Histological assessments further showed abundant inflammatory cell infiltration and elevated numbers of TRAP^+ve^ osteoclasts on the bone surface (**Figures 6B, E**). On the other hand, Preg treatment (both low and high doses) significantly lessened the destructive effects of LPS, reducing inflammatory cell infiltration and osteoclast activity. In particular, treatment with high dose Preg (10 mg/kg bodyweight) almost completely prevented LPS-induced bone destruction and bone loss, with bone volume almost comparable to that of sham controls (**Figures 6A, C**). The number of TRAP^+ve^ osteoclasts in high dose Preg treated group were also comparable to sham controls indicating the inhibition of osteoclast formation and therefore activity (**Figure 6E**).

### Preg Does Not Affect Osteoblast Differentiation and Mineralization

**Figure 7:**
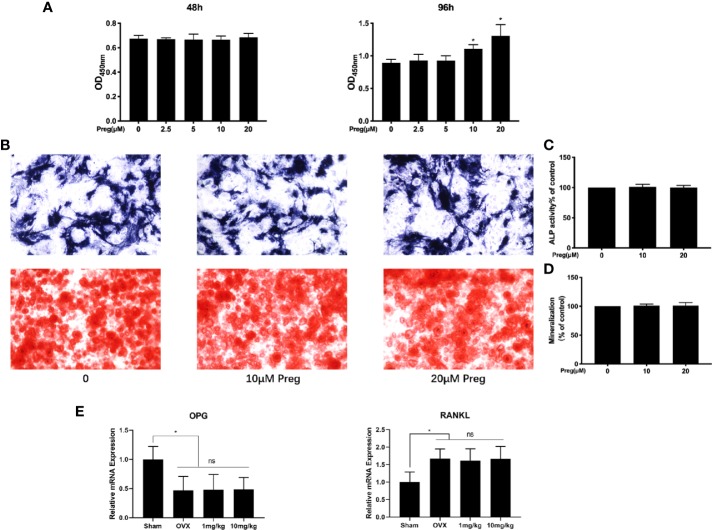
Pregnenolone (Preg) does not no affect the osteogenic differentiation and mineralization of BMSC-derived osteoblasts. **(A)** BMSC cell viability as assessed by CCK-8 assay following treatment without or with indicated concentrations of Preg for 48 and 96 h. **(B)** ALP and alizarin red S staining of BMSC-derived osteoblasts following osteogenic differentiation without or with 10 and 20 μM Preg for 7 and 21 d respectively. **(C, D)** ALP and mineralization activity relative to untreated controls were quantified. **(E)** The relative expression of OPG and RANKL in bone samples were quantified by real time PCR. Values presented as the mean ± standard deviation (n = 3); *p < 0.05.

Maintenance of bone homeostasis requires the balance but opposing functions of bone resorption by osteoclasts and bone formation by osteoblasts. Thus we examined whether Preg affects osteoblast differentiation and mineralization function. To this end, primary BMSCs were extracted from mice long bones and was first subjected to cell viability/cytotoxicity analysis as performed for BMMs. As shown in **Figure 7A**, treatment of BMSCs with indicated concentrations of Preg did not affect cell viability. Interestingly, at 96 h, significant elevation in cell proliferation was observed at 10 and 20 μM of Preg treatment. However, when BMSCs were cultured under osteogenic condition in the presence of 10 or 20 μM of Preg, no change was observed in alkaline phosphatase activity 7 d after osteogenic differentiation (**Figures 7B, C**). Similarly Preg treatment did not inhibit nor enhance mineralization activity after 21 d of osteogenic differentiation (**Figures 7B, D**). Next, we tested the expression of OPG and RANKL *in vivo*, and there was no significant difference regarding the expression of OPG and RANKL between the OVX and Preg treatment groups (**Figure 7E**). Thus despite enhancing BMSC cell proliferation at high doses, Preg does appear to enhance (or inhibit) osteogenic differentiation or mineralization activity of BMSCs.

### Preg Prevented Systemic OVX-Induced Bone Loss *In Vivo*

**Figure 8:**
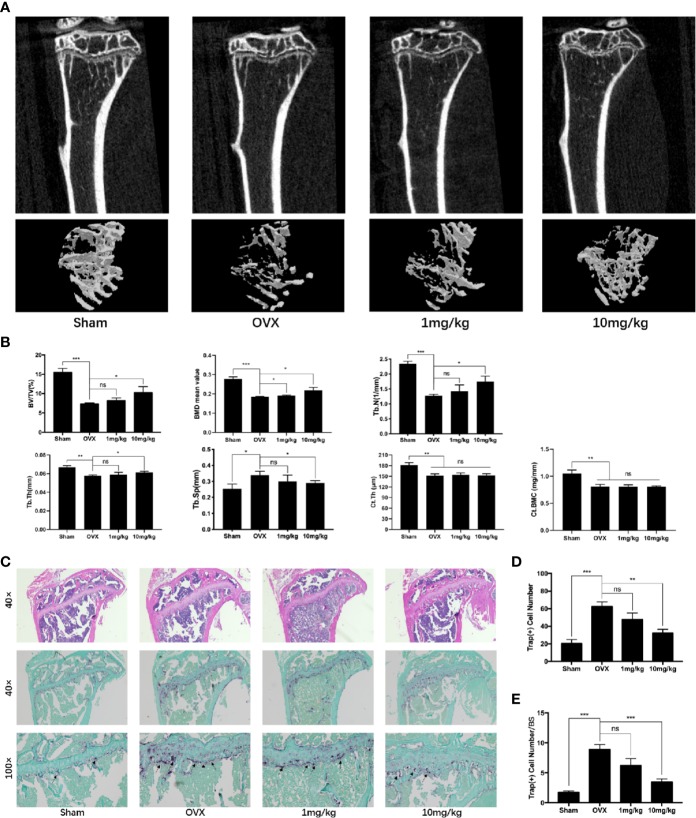
Pregnenolone (Preg) prevents bone loss in ovariectomized (OVX) mice *in vivo*. **(A)** Representative 3D μCT reconstructions of mouse tibial bone from sham (PBS injection), OVX (PBS injection), OVX with 1 mg/kg Preg (low dose), and OVX with 10 mg/kg Preg (high dose). **(B)** Quantitative bone morphometric parameters of bone volume to total tissue volume (BV/TV), bone surface to tissue volume (BS/TV, mm^−1^), trabecular spacing (Tb.Sp., mm), trabecular number (Tb.N., mm^−1^), cortical thickness (Ct.Th), and cortical bone mineral content (Ct.BMC) were measured. **(C)** Representative histological assessment of tibial bone sections stained for H&E and TRAP activity (40X and 100X magnification) to assess osteoclast activity. Tibial bone samples were decalcified in 10% EDTA for 2 weeks, embedded in paraffin blocks, sectioned and then stained with H&E and TRAP. **(D, E)** Quantitative assessment of **(D)** the total number of TRAP^+ve^ cells and **(E)** the number of osteoclast per bone surface were conducted. Values presented as the mean ± standard deviation (n = 3); *p < 0.05, **p < 0.01, ***p < 0.001.

Apart from the local suppressive effect of Preg on osteolysis, we further investigated whether Preg can be systemically used to prevent bone loss. Similar protective effects were seen in the OVX-induced bone loss model but only when mice were given high-dose of Preg (10 mg/kg bodyweight). No significant protective effects were observed when mice were given low dose of Preg (1 mg/kg bodyweight) over the 4 week experimental period (**Figure 8**). Three-dimensional reconstructions of the tibial bone tissue shows significant reduction in bone volume and trabecular bone loss after bilateral ovariectomy (**Figures 8A, B**). TRAP staining of the tibial bone sections shows significant enhancement in the total number of TRAP^+ve^ osteoclasts and the number of osteoclasts lining the trabecular bone surface (**Figures 8C–E**). However, treatment with low dose Preg did not protect against OVX-induced bone loss and trabecular bone deterioration. Only at high doses of 10 mg/kg did Preg demonstrate its protective effect against OVX-induced reduction in trabecular bone *via* the suppression of osteoclast formation and activity. In addition, there was no significant difference in cortical bone thickness between OVX and Preg treatment groups. (**Figure 8B**). Thus collectively, and in general, Preg particular at high doses demonstrates protective against inflammatory bone destruction as well as postmenopausal bone loss *via* the inhibition of osteoclast formation and bone resorption. It is worth noting that no adverse effects were observed in mice treated with high dose Preg suggesting that Preg may exhibit a relatively safe *in vivo* drug profile.

## Discussion

Bone homeostasis is maintained by the balanced and coordinated activities of osteoclastic bone resorption and osteoblastic bone formation. Steroid hormones including sex hormones have a dramatic impact on bone growth, remodeling, and maintenance throughout life. Hence alterations in steroid hormone levels are important factors contributing to the development of many osteolytic bone diseases including post-menopausal osteoporosis and inflammatory bone destruction (Manolagas, 2013). For example, estrogen deficiency which leads to elevated osteoclast formation and activity is one of the major underlying causes of post-menopausal osteoporosis (Riggs et al., 1998; Raisz, 2005). Similarly declining androgen levels cause bone loss in men and increases bone remodeling (Katznelson et al., 1996; Daniell, 1997). Preg is an endogenous steroid and the grand precursor of almost all of the other steroid hormones including estrogen, progesterone, testosterone, and glucocorticoids, and mineralocorticoids (Henderson et al., 1950; Berg et al., 2002; Marx et al., 2011). As such is Preg has been termed the “mother or grandmother” hormone. Like estrogen, the level of Preg dramatically decline with age, with the levels at the age of 75 being 60% less than the levels produced in the mid 30s (Roberts, 1995; Morley et al., 1997). It has been proposed that Preg has potential as a novel anti-osteoporotic agent (Maurya et al., 2017). In our study we provided further evidence to support this claim. Preg was found to protect mice against both LPS-induced inflammatory bone destruction as well as OVX-induced bone loss *in vivo via* the inhibition of osteoclast formation and bone resorptive activity. *In vitro* cellular assays further confirmed a direct inhibitory effect of Preg on RANKL-induced osteoclast formation from primary BMMs and also mature BMM-derived osteoclast bone resorption. Our study did not find any effect of Preg on the osteogenic differentiation of BMSCs or downstream mineralization activity.

Induction of osteoclast formation involves the timely and efficient activation of various signaling cascades in monocytic precursor cells. Two key cytokines, M-CSF and RANKL, act in concert to regulate the activation of these signaling cascades that leads to monocytic cell differentiation, cytoskeletal rearrangement, cell fusion and maturation, and subsequent mature osteoclast bone resorption and survival. The NF-κB and MAPK (ERK, p38, and JNK) signaling cascades are among the earliest activated following binding of RANKL to receptor RANK. The canonical activation of NF-κB involves the rapid phosphorylation and proteasomal degradation of IκB proteins, which in turn allow for phosphorylation and nuclear translocation of unbound NF-κB p65 subunits, where it functions to transcriptionally activate target genes. Similarly, the MAPK signaling triad of ERK, p38 and JNK are activated by phosphorylation which enables nuclear translocation and binding to respective DNA response elements to cooperatively activate transcription of target genes. Genetic deletion or pharmacological inhibition these signaling cascades have been shown to arrest osteoclast formation and bone resorption (Karakawa et al., 2009; Kameda et al., 2013; Mediero et al., 2013; Ma et al., 2015; Jiao et al., 2017; Kim et al., 2018). The efficient early activation NF-κB and MAPK is necessary for the downstream induction and upregulation of transcription factors c-Fos and NFATc1. Importantly, NFATc1 has been regarded as the master transcription factor for osteoclast formation and function, transcriptionally regulating the expression of various osteoclast genes including *DC-STAMP*, *ATP6V0d2*, *TRAP*, *CTSK*, and *NFATc1* itself (Kim et al., 2008; Kim et al., 2013; Liu et al., 2016; Chiu et al., 2017; Zeng et al., 2017; Pang et al., 2019). As with the loss of early signaling events, the lack of either c-Fos or NFATc1 results in abolishment of osteoclast formation leading to increase bone mass and osteopetrosis in mice (Asagiri et al., 2005; Arai et al., 2012; Toray et al., 2017). Using biochemical western blot assays, we showed that Preg treatment resulted in almost complete attenuation of RANKL-induced phosphorylation of ERK as well as marked inhibition of IκBα degradation and phosphorylation of p65. The inhibition of these early signaling cascades by Preg subsequently reduced the efficient induction of c-Fos and NFATc1. The attenuated induction of NFATc1 is line with decreased expression of osteoclast marker genes. Thus the anti-osteoclastogenic effects of Preg can in part attributed to the inhibition of ERK and NF-κB signaling and consequently the induction of c-Fos and NFATc1.

ROS have also been shown to activate osteoclast formation and activity *via* the regulation of signaling cascades (Garrett et al., 1990; Bax et al., 1992). Signaling cascades shown to be responsive to ROS levels include NF-κB, PI3K, and the MAPK’s JNK and p38 (Schreck et al., 1991; Finkel and Holbrook, 2000; Droge, 2002; Paolicchi et al., 2002). Treatment of BMMs with anti-oxidants was shown to decrease in RANKL-induced IκBα phosphorylation and degradation, as well as phosphorylation of MAPK members (Ha et al., 2004; Lee et al., 2005). Furthermore, numerous studies have shown dramatic reduction in anti-oxidant defenses in post-menopausal osteoporotic patients and mouse models of estrogen-deficiency osteoporosis, resulting in markedly elevated ROS levels and bone loss (Dou et al., 2016; Liu et al., 2019). In previous studies, it has been confirmed that Preg can promote the production of ROS (Li et al., 2017). However, in our studies, we showed that Preg treatment significantly reduced intracellular ROS levels during RANKL-induced osteoclast formation. To our knowledge, our study is the first to show that Preg exhibits potent anti-oxidant properties, and the effect of Preg on RANKL signaling cascade may involve the suppression of ROS induction.

Despite these interesting findings, the precise mechanism by which Preg exerts its biological effects in not well understood. In general, steroid hormones exert their biological effects by binding their intracellular receptors. As the grand precursor steroid, Preg, the mechanism of action is likely similar. However, a specific receptor for Preg has yet to be identified. Studies have shown that Preg can acts as an modulator of NMDA receptor and as an agonist of pregnane X receptor (PXR) (Malayev et al., 2002; Shizu et al., 2013; Smith et al., 2014; Chopra et al., 2015). Interestingly, both NMDA receptor and PXR have been shown to play a role in osteoclast formation and function. Activated NMDA receptors have been shown to involve in the induction of osteoclast formation *via* the NF-κB signaling pathway (Merle et al., 2003). Furthermore, specific antagonists of NMDA receptor inhibited osteoclast formation, osteoclast sealing zone formation and bone resorption (Merle et al., 2003). Likewise, PXR knockout mice was shown to exhibit osteopenia with reduced bone formation and enhanced bone resorption. Additionally, meclizine, a PXR agonists was found to prevent OVX-induced bone loss by inhibiting osteoclast formation. Mechanistically meclizine attenuated RANKL-induced activation of NF-κB, ERK and p38, and subsequent c-Fos and NFATc1 induction *via* PXR-dependent mechanism (Guo et al., 2017). Based on these data, it is tempting to suggest that Preg acts *via* both NMDA receptor (as an antagonist) and PXR (as an agonist) to exert its anti-osteoclastogenic and anti-resorptive effects. Further investigation is needed to verify this hypothesis. However, given that Preg serves as the grand precursor to almost all steroid hormones, we cannot rule out the possibility that the effects seen in our study is the result of the intracellular conversion of Preg into downstream effecter steroid hormones such as estrogen, which is renowned for its anti-osteoclastogenic and anti-resorptive effects. Such possibility will be topic of future investigations.

In all, our current study provides some interesting finding for the biological effects of Preg on osteoclast formation and activity. Preg was found to inhibit RANKL-induced differentiation of primary BMMs into multinucleated osteoclasts and subsequent downstream bone resorptive activity *in vitro* and prevented osteoclast-mediated inflammatory bone destruction and estrogen-deficiency induced bone loss *in vivo*. The anti-osteoclastogenic effect of Preg could be in part attributed to the suppression of RANKL-induced ROS generation and activation of ERK and NF-κB. This consequently attenuated the induction of c-Fos and NFATc1, key transcription factors for osteoclast formation and function. These results provide some promising evidence for the use of Preg in the prevention or therapeutic treatment of osteoclast-mediated osteolytic bone diseases.

## Data Availability Statement

The raw data supporting the conclusions of this article will be made available by the authors, without undue reservation, to any qualified researcher.

## Ethics Statement

The animal study was reviewed and approved by Animal Care Committee of Guangxi Medical University.

## Author Contributions

XS and CZ mainly carried out the studies, were involved in the experimental design and statistical analysis, and drafted the manuscript. XS and CZ performed cell culture and participated in all vitro and vivo experiments. HG, JC, YT, and FW were involved in the histological examination of tissue samples. QL and XL were involved in the sample collection and statistical analysis. AQ and LS conceived the study, involved in its design and coordination, and helped to draft the manuscript. All authors read and approved the final manuscript.

## Funding

This research is supported by grants from the Natural Science Foundation of China (No.81772373, No.81572167, No.81703506), by the Shanghai Municipal Education Commission —Gaofeng Clinical Medicine Grant Support, and by the SHIPM-pi fund (No. JY201804 & No.JC201801) from Shanghai Institute of Precision Medicine, Ninth People`s Hospital Shanghai Jiao Tong University School of Medicine.

## Conflict of Interest

The authors declare that the research was conducted in the absence of any commercial or financial relationships that could be construed as a potential conflict of interest.
